# Increasing and sustaining discharges by noon – a multi-year process improvement project

**DOI:** 10.1186/s12913-024-10960-x

**Published:** 2024-04-17

**Authors:** Ryan Bailey, Ankur Segon, Sean Garcia, Saket Kottewar, Ting Lu, Nelson Tuazon, Lisa Sanchez, Jonathan A. Gelfond, Gregory Bowling

**Affiliations:** 1 The University of Texas Health Science Center at San Antonio, 7703 Floyd Curl Dr, San Antonio, TX 78229 USA; 2 University Health, 4502 Medical Drive, San Antonio, TX 78229 USA

**Keywords:** Patient discharge, Hospitalist, Length of stay, Patient readmission, Hospital capacity, Multidisciplinary, Patient safety, Process improvement, Emergency department boarding

## Abstract

High hospital occupancy degrades emergency department performance by increasing wait times, decreasing patient satisfaction, and increasing patient morbidity and mortality. Late discharges contribute to high hospital occupancy by increasing emergency department (ED) patient length of stay (LOS). We share our experience with increasing and sustaining early discharges at a 650-bed academic medical center in the United States. Our process improvement project followed the Institute of Medicine Model for Improvement of successive Plan‒Do‒Study‒Act cycles. We implemented multiple iterative interventions over 41 months. As a result, the proportion of discharge orders before 10 am increased from 8.7% at baseline to 22.2% (*p* < 0.001), and the proportion of discharges by noon (DBN) increased from 9.5% to 26.8% (*p* < 0.001). There was no increase in balancing metrics because of our interventions. RA-LOS (Risk Adjusted Length Of Stay) decreased from 1.16 to 1.09 (*p* = 0.01), RA-Mortality decreased from 0.65 to 0.61 (*p* = 0.62) and RA-Readmissions decreased from 0.92 to 0.74 (*p* < 0.001). Our study provides a roadmap to large academic facilities to increase and sustain the proportion of patients discharged by noon without negatively impacting LOS, 30-day readmissions, and mortality. Continuous performance evaluation, adaptability to changing resources, multidisciplinary engagement, and institutional buy-in were crucial drivers of our success.

**Supplementary Information:**

The online version contains supplementary material available at 10.1186/s12913-024-10960-x.

## What is already known on this topic

Discharge before noon (DBN) has been successfully implemented by multiple institutions with the goal of alleviating high hospital occupancy and emergency department boarding.

## What this study adds

This study demonstrates the feasibility and sustainability of DBN over an extended period of 41 months despite numerous barriers, including multiple patient surges due to COVID-19.

## How this study might affect research, practice, or policy

We provide our methods and experience as a guide for the practical implementation of processes to achieve and sustain DBN.

## Introduction

Discharge before noon (DBN) is a key intervention for providing safe and timely patient care given skyrocketing hospital occupancy and emergency department (ED) boarding. High hospital occupancy increases boarding times for patients in the ED, which in turn leads to ED overcrowding [1, 2]. ED overcrowding increases patient morbidity and mortality, prolongs wait times for patients, decreases patient satisfaction and is associated with physical injury to ED providers [1, 3, 4]. Early discharges from the inpatient setting can help alleviate ED boarding by reducing ED LOS. There is a nationwide focus on improving the proportion of early discharges in the United States.

Several studies from across the world have demonstrated mixed effects of early discharges on quality metrics [5–12]. Two studies in the United States showed that a multidisciplinary DBN intervention reduced the observed to expected (O/E) length of stay as well as 30-day readmission rates [5, 6]. Additionally, an increase in the proportion of discharges by noon (DBNs) is associated with earlier and evenly spread-out arrival of admissions on inpatient floors from the ED [7]. Khanna et al. showed that early discharges helped reduce occupancy levels and overcrowding in Australian health settings [8]. Another study in Lebanon used Six Sigma methodology to discharge patients earlier in the day and successfully improved hospital LOS and ED mean LOS for admitted patients [9]. All these studies report the impact of their DBN interventions over a relatively short period of several months.

Other studies have shown a more mixed impact of DBN initiatives on LOS. It has been postulated that emphasis on DBN may inadvertently increase LOS by converting a potential late in the day discharge to an early discharge on the subsequent day. While some studies have found no association between DBN and LOS, others have reported an increase in LOS with DBN initiatives [5, 10–12]. The impact of DBN on patient safety outcomes such as mortality and readmission rates is also unclear.

At our institution, high hospital occupancy and ED boarding led to our DBN initiative. We share our experience in increasing and sustaining early discharges at our institution over a period of forty-one months, including the period impacted by the COVID-19 pandemic. We also report our performance on balancing metrics such as LOS, readmission rates and mortality rates over the same period.

## Methods

### Setting

This multidisciplinary project was sequentially executed in multiple adult inpatient units at our 650-bed academic level 1 trauma center and safety net hospital in Texas, United States. We have a total of 14 inpatient hospital medicine teams caring for an average daily census of 210 patients. Six of these teams are teaching teams and 8 are direct care teams.

This project was executed over 3 phases. Our hospital medicine teams are geographically dispersed across 7 different inpatient units in 3 different towers. Each teaching team is composed of a hospitalist attending physician, a senior resident (Post-Graduate Year 2 or 3 [PGY-2 or PGY-3]), two interns (PGY-1), and two third-year medical students. Each direct care team is composed of an attending hospitalist physician and an advanced practice provider (APP).

### Interventions

Our project spanned 3 phases, and collectively the 3 phases described make up the post-intervention period. Interventions were targeted at the 6 internal medicine teaching teams during phase 1. We chose to start with our teaching teams, as most of their patients were geographically located on the same unit. We expanded this DBN initiative to each of our 8 internal medicine direct care teams in phase 2. We used the Institute of Healthcare Improvement (IHI) Model For Improvement (MFI) to design and implement interventions using sequential Plan‒Do‒Study‒Act (PDSA) cycles [13]. A multidisciplinary team of hospitalist attending physicians and APPs, internal medicine resident physicians, case managers, social workers and nurses finalized, implemented, and assessed each cycle of change. Successive interventions during each phase were identified based on a review of our DBN performance and feedback from all members of the team. This project was sponsored by the Chief Operating Officer of the Hospital. The Chief Nursing Officer and vice president of case management actively participated in all PDSA cycles. Multiple needs assessment meetings were carried out with frontline hospitalists, nurses, case managers and social workers before phase 1 and in-between the 3 phases. Key interventions in each phase are described below and summarized in Table 1.

**Table 1:** Table of interventions/events during the DBN initiative

Phase	Intervention	Subcategory	Description	Date
**Phase 1**	**Intervention launched for first teaching teams**	Main intervention Infrastructure	Interdisciplinary huddle with nurses, physicians, and case management to proactively plan discharges	12/01/18
	**Intervention launched for remaining teaching teams on other floors**	Main intervention Infrastructure		01/01/19
**Phase 2**	**Discharge Expediters & Case Management added to direct care medical teams**	Infrastructure	Team-based care coordination	07/01/19
	**DBN initiative work group created to include physicians and APP's**	Fostering collaboration on care teams	Physicians and APPs reflect on past performance and opportunities to improve early discharges	05/21/20
	**Faculty Development Conference for physician and APP groups regarding DBN initiative**	Educating house staff and faculty Fostering collaboration on care teams One-time event	Event by DBN initiative Champions to teach physician and APP staff best practices for early discharge	05/28/20
	**New Post Graduate Year 1 (PGY-1) residents are inducted into the program**	Obstacles to achieving high DBN rates	PGY-1 s require time to acclimate to residency and the DBN initiative	07/01/20
	**Large COVID Case Surge**	Obstacles to achieving high DBN rates	COVID Infection rates soar in early July, crowding the hospital and ED	07/01/20
	**Epic (Electronic Health Records) Rollout**	Infrastructure Obstacles to achieving high DBN rates	All providers required time to adapt to new EHR leading to a barrier to early discharge	07/11/20
	**Implementation of a discharge checklist for teaching teams**	Educating house staff and faculty	Steps to prepare a patient for discharge	10/27/20—11/17/20
	**Department administrator sends emails to physicians and APP's recognizing high performers**	Direct engagement with practitioners	Feedback for providers regarding early discharge performance only for the highest performing providers	11/20/20
	**DBN initiative Noon conference for residents and presentation of Discharge Checklist**	Educating house staff and faculty One-time event	Hospitalist teaching house staff the steps to prepare a patient for discharge	12/09/20
	**Email to teaching team attending physicians each block reminding them of the DBN Initiative**	Educating house staff and faculty Direct engagement with practitioners	Email includes best practices for early discharge	12/10/20
	**Projects in Progress Faculty Development conference**	Educating house staff and faculty Direct engagement with practitioners	Small meeting with DBN initiative champions and hospitalists to rebuild interest in early discharge	04/27/21
	**Medical teams switch from a team-based to unit-based case management staffing model**	Staffing Model Infrastructure Obstacles to achieving high DBN rates	Increases the number of case managers with whom providers must interact; prevents in-person interdisciplinary flash rounds	06/15/21
**Phase 3**	**New Post Graduate Year 1 (PGY-1) residents are inducted into the program**	Obstacles to achieving high DBN rates	PGY-1s require time to acclimate to residency	07/01/21
	**Large COVID Case Surge**	Obstacles to achieving high DBN rates	COVID Infection rates soar in early July, crowding the hospital and ED	07/01/21
	**Began sharing DBN data to individual practitioners via performance packets**	Educating house staff and faculty Direct engagement with practitioners	All providers receive feedback rather than exclusively top performers	07/15/21
	**Implementation of Electronic Secure Chat with Case Management and Nursing**	Infrastructure	Substitute for in-person interdisciplinary flash rounds; occurs each morning. Allows practitioners to remotely discuss patient readiness for discharge in a algorithmic manner	07/15/21
	**Presentation of DBN initiative to residents**	Educating house staff and faculty One-time event	Education for all house staff to promote DBN initiative on teaching teams	08/18/21

### Phase 1: December 2018-June 2019

#### Team-building workshops

Each teaching team had a hospitalist attending physician champion selected to join a multidisciplinary team of nursing and care coordination leaders to plan the launch of the intervention. Checklists were developed for providers, nursing, and case management to prepare for discharge. These checklists as well as a discharge process map are available in the online Supplemental materials. Each teaching team had a nurse case manager (CM) or social worker (SW) assigned. Daily afternoon multidisciplinary rounds were set up to anticipate and prepare for discharges the following morning, and the structure and responsibilities for these rounds were established and agreed upon by all team members. These multidisciplinary rounds were christened “flash rounds” to emphasize their concise duration of under 10 min.

### Afternoon flash rounds

Providers, case management, and nursing leadership from each medical team met daily at 2 pm for flash rounds. Flash rounds were scripted, and four key questions were rapidly reviewed for each patient: (1) Why is the patient admitted to the hospital? (2) Why is the patient still in the hospital? (3) What barriers are there to a safe discharge? (4) Where will the patient go after discharge? A stoplight system was used to reflect a patient’s readiness for discharge. A green patient is expected to be discharged the same day, a yellow patient is anticipated to be discharged within 24–48 h, and a red patient is not anticipated to be discharged in the next 48 h. Obstacles to discharge identified during flash rounds are assigned to a team member (provider, nursing, or case management) for escalation. Examples of escalation include case management contacting radiology administrative leadership to get an interventional radiology procedure completed without delay and nursing contacting Physical or Occupational Therapy to assist with final recommendations for post-acute care.

### Morning discharge huddles

Each morning, case management and nursing leadership on the floor briefly huddled with the teaching team to identify green and yellow patients based on flash rounds discussion from the day prior. This allows the identification of any last-minute obstacles to optimize early discharges. Nursing and case management immediately escalate any remaining barriers and proactively communicate with the providers to ensure timely entry of a discharge order, with a goal for order entry before 10 am to allow discharge by noon.

### Nursing shift huddles

At each nursing shift change, there is intentional discussion of green and yellow patients as part of a standardized handoff. At the evening shift change, day shift nurses highlight yellow patients who may be green the following day based on discussion during flash rounds and the patient’s clinical progression. The night shift nurses review the discharge readiness checklist to prepare patients for early discharge in the coming day shift. Each morning at handoff, the night shift nurse highlights green patients for the day shift nurse. Standardized checklists for green and yellow patients are available in the Supplemental materials.

### Phase 2: July 2019-June 2021

#### Expansion of intervention

After the rollout was complete for teaching teams, the intervention was expanded to direct care teams with physician/APP team members. To facilitate this, each direct care team had support from care coordination (CC) that included a case manager (CM) or social worker (SW), and a discharge expediter (DE). Roles were clearly established for CMs, SWs, DEs and providers. Flash rounds on direct care teams are held each afternoon along with pre-rounding morning discharge huddles described above. Participants include physician/APP providers on the team along with the assigned CM and DE. The CM communicates with nursing regarding discharge preparations, and the CM and DE assist with escalations to avoid any discharge delays.

### Feedback to providers

Feedback was provided in various forms after the intervention was launched on teaching teams and then direct care teams. For teaching teams, our hospital medicine administrative staff took “goody bags” to recognize high-performing residents and interns in person. A meal voucher was provided to residents by the unit clerk whenever a discharge order was placed before 9 am. We awarded stars to high-performing hospitalist physicians on direct care teams. Beginning November 2020, we began sending feedback to both APPs and physicians in a weekly email. The physician leadership of our Hospitalist group recognizes top performing individuals publicly through responses to these emails.

### Phase 3: July 2021-May 2022

#### Leadership discharge rounds

Beginning in July 2021, leaders from nursing, care coordination, and bed planning and the hospitalist group started participating in early afternoon calls to review discharges each day. Each nursing unit leader reports the total number of discharges, number of discharges before noon, and reviews any remaining green patients who are still pending discharge. Barriers to discharge are discussed, and any feasible escalation is done at the time of this call to facilitate remaining discharges.

### Electronic secure-chat communication

In June 2021, the combination of case management shifting to a unit-based rather than team-based model due to staffing limitations and a new surge in COVID-19 infections led to the elimination of flash rounds and morning discharge huddles and a decline in early discharges (Fig. 2). To respond to this challenge, our next improvement cycle included the creation of virtual flash rounds using our Electronic Health Record (Epic™). Epic supports real-time group chatting capability via “secure chat” [14]. Providers initiated a “secure chat” with case management to discuss their patients. Case managers added the patient’s bedside nurse to the secure chat. Case management also communicated with nursing leadership on the floor to escalate any obstacles to discharge.

### Data collection and statistical analysis

#### Primary outcome metrics

Primary outcome metrics include the percentage of Discharge Orders placed before 10 am (DCOB) and the percentage of completed Discharges By Noon (DBN). These data are sourced from the electronic medical record. We tracked baseline data for the 11 months leading up to the launch of the intervention in December 2018 and for 41 months following the launch of the first phase in January 2019. The change in proportion of discharge orders placed by 10 am and discharges by noon are presented over time using Statistical Process Control (SPC) charts. A run of 6 or more consecutive points on either side of the mean on an SPC chart is called a shift and indicates that the observed change is a result of systematic interventions as opposed to random variation [15].

We compared DCOB and DBN between baseline and all 3 PDSA cycles combined, and between baseline and PDSA 1, PDSA 1 and 2, and PDSA 2 and 3. Data were tested for normality using Shapiro Wilk tests. Welch’s two sample t-tests were used to compare groups if data were normally distributed, while Wilcoxon rank sum tests were used if data were not distributed normally. In addition, we performed an interrupted time series analysis of DCOB and DBN data. The monthly counts of DCOB and DBN were totaled for all years. This count was modeled as an interrupted time series with a generalized, linear mixed effects model with a random effect for year and a logit link. In addition to the period effect (Baseline, Phase 1, 2, 3), one month lagged outcomes were included as autoregressive covariate terms (LagDCOB and LagDBN). Changes in DCOB and DBN within the baseline period and phases 1, 2 and 3 were first modeled by calculating period specific slopes while changes between baseline period and phases 1,2 and 3 were modeled as intercepts. For the DCO outcome, a period specific slope was used to account for trends within a period. This slope was not included for the DBN outcome in the final model because there were no significant trends. The Baseline period did not have significant slope for either DCO or DBN and was excluded from the models. All modeling was done using R v4.3 (Vienna, Austria), the generalized mixed effects model was implemented with the *lme4* package, and a two-sided significance level of 0.05 was used throughout.

### Balancing metrics

Key balancing metrics include risk-adjusted length of stay (RA-LOS), risk-adjusted 30-day readmissions (RA-readmissions), and risk-adjusted mortality (RA-mortality). Our institution utilizes the MIDAS (Meaningful Integration of Data Analytics and Services) platform to gather information from our electronic health record (EPIC™). MIDAS uses proprietary methodology to calculate expected values for LOS, 30-day readmissions, and mortality, utilizing information including facility, patient demographics (sex, age), admission source, admitting service, acute care status (inpatient versus observation), discharge disposition, International Classification of Diseases (ICD)-9 and ICD-10 diagnoses and procedures, Medicare Severity Diagnosis Related Groups (MS-DRG) medical/surgical classification, DRG relative weight, and total charges [16]. The MIDAS platform pulls observed values from our health record to pair with expected values and calculates Observed to Expected (O/E) ratios for each metric, yielding reports of RA-LOS, RA-readmissions, and RA-mortality. We compared O/E ratios for RA-LOS, RA-readmissions and RA-mortality between baseline and phases 1, 2 and 3 combined. Data were tested for normality using Shapiro Wilk tests. Welch’s two sample t-tests were used to compare groups if data were normally distributed, while Wilcoxon rank sum tests were used if data were not distributed normally.

The study was approved locally as a quality improvement initiative and was deemed exempt from traditional institutional review board approval.

## Results

Key interventions and contextual events impacting the quality improvement initiative are outlined in Table 1.

Between Baseline and Phase 1, the average Rate of Discharge Orders Before 10 am (DCOB) increased from 8.7% (741/8514) to 15.1% (399/2566) ( *p* < 0.001). Between Phase 1 and Phase 2, the average DCOB increased from 14.4% (617/4228) to 22.6% (3302/14545) (, *p* < 0.001). Between Phase 2 and Phase 3, the average DCOB increased from 22.6% (3302/14545) to 29.4% (2311/7917)( *p* = 0.001). Finally, between Baseline and all three phases combined, the average DCOB increased from 8.7% to 22.2% (7330/33518) ( *p* < 0.001).

Between Baseline and Phase 1, the average Rate of Discharges Before 12 pm (DBN) increased from 9.5% (808/8514) to 26.8% (8746/32591) ( *p* < 0.001). Between Phase 1 and Phase 2, the average DBN increased from 26.7% (633/2566) to 27% (3952/14545) (*p* = 0.81). Between Phase 2 and Phase 3, the average DBN increased from 27% (3952/14545) to 27.2% (2139/7917) (*p* = 0.91). Finally, between Baseline and all three phases combined, the average DBN increased from 9.5% (808/8514) to 26.8% (8746/32591) (*p* < 0.001).

**Fig. 1:**
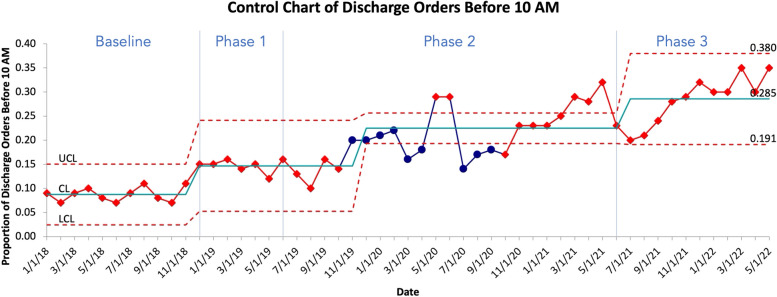
Control chart of average calendar month DCOB percentage for all medical units. Data points in red indicate non-random variation. UCL indicates Upper Control Limit, CL indicates Central Limit (mean), LCL indicates Lower Control Limit

**Fig. 2:**
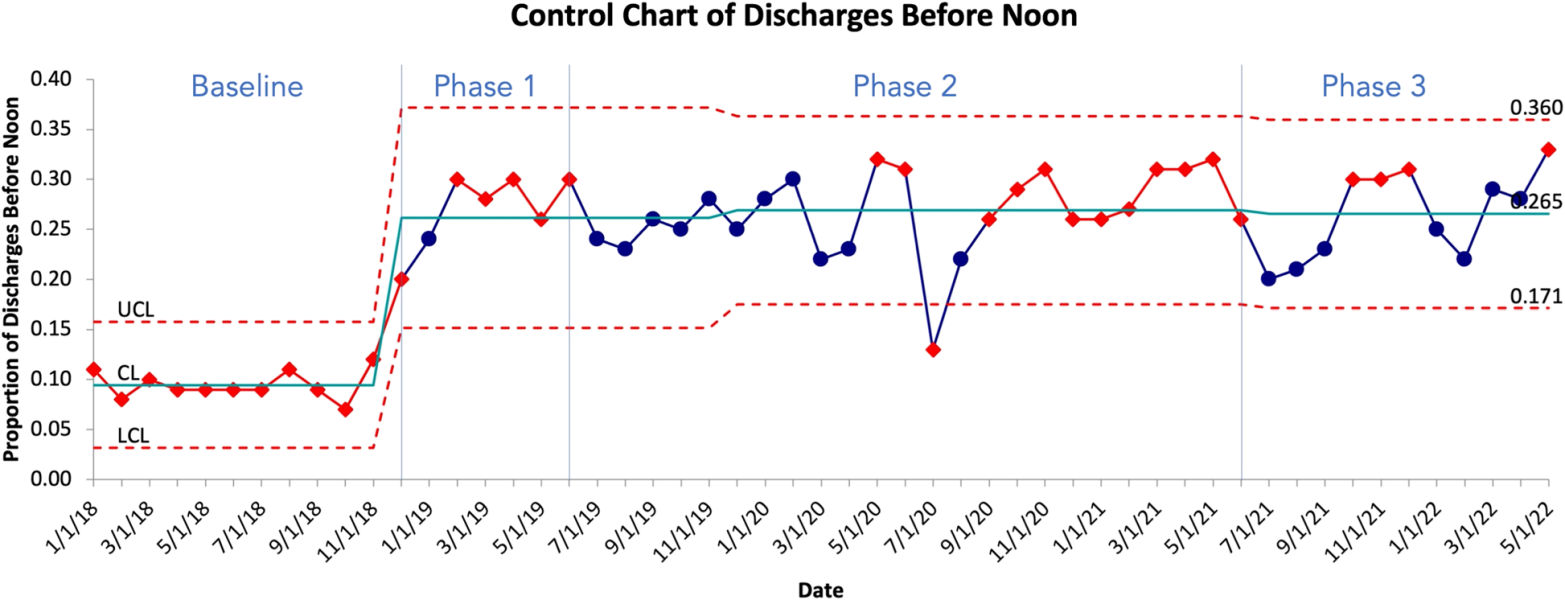
Control chart of average calendar month DBN percentage for all medical units. Data points in red indicate non-random variation. UCL indicates Upper Control Limit, CL indicates Central Limit (mean), LCL indicates Lower Control Limit

Figures 1 and 2 show control charts of DCOB and DBN. Data points shown in red indicate a nonrandom change in performance on DCOB and DBN following our interventions that cannot be accounted for by inherent randomness in the system [15].

Table 2 shows a statistically significant improvement in DBN between baseline period and phase 1 (OR 2.2, *p* < 0.001), phase 2 (OR 2.2, *p* < 0.001, and phase 3 (OR 2.5, *p* < 0.001).

**Table 2:** Change in DBN within baseline period (slope) and difference in DBN between baseline and phases 1, 2 and 3 (intercepts)

**Characteristic**	**OR**^*1*^	**95% CI**^a^	***p*-value**
Baseline Slope	0.93	0.65, 1.34	0.7
Phase 1 Intercept	2.22	1.72, 2.85	< 0.001
Phase 2 Intercept	2.23	1.79, 2.77	< 0.001
Phase 3 Intercept	2.48	1.94, 3.16	< 0.001

^a^*OR* Odds Ratio, *CI* Confidence Interval

Table 3 shows that the rate of DCO before 10 am increased from baseline to Phase 1 (OR = 1.49, 95% CI 1.01, 2.20; *p* = 0.046). Though Phase 1 was improved over Baseline, the improvement was in the beginning of Phase 1 and did not continue throughout Period 1 until Phase 2. The Period specific slopes are significant except for Phase 1, indicating improvement of the DCO throughout Phase 2 (OR per year 1.92, 95% CI 1.49, 2.49; *p* = < 0.001) and Phase 3 (OR per year 1.88, 95% CI 1.43, 2.48; *p* = < 0.001).

**Table 3:** Change in DCOB within baseline, and phases 1, 2 and 3 (slope) and difference in DCOB between baseline and phases 1, 2 and 3 (intercepts)

**Characteristic**	**OR**^a^	**95% CI**^a^	***p*-value**
Baseline Slope	0.80	0.48, 1.33	0.4
Phase 1 Intercept	1.49	1.01, 2.20	0.046
Phase 1 Slope	0.80	0.52, 1.21	0.3
Phase 2 Intercept	1.38	0.90, 2.13	0.14
Phase 2 Slope	1.92	1.49, 2.49	< 0.001
Phase 3 Intercept	5.54	2.86, 10.7	< 0.001
Phase 3 Slope	1.88	1.43, 2.48	< 0.001

^a^*OR* Odds Ratio, *CI* Confidence Interval

Between baseline and all three phases of the intervention period, there was a slight decrease in RA-LOS and RA-readmissions and no change in RA-mortality (Table 4).

**Table 4:** Change in balancing metrics between Baseline (pre-intervention) and post-intervention

Variable	Baseline	Post-Intervention	Percent Change	*P* value
RA-LOS	1.16	1.09	-6.01%	0.01
RA-Mortality	0.65	0.61	-6.31%	0.62
RA-Readmissions	0.92	0.74	-19.52%	0.0006

*RA* Risk Adjusted

*p*-value < 0.05 is statistically significant

**Fig. 3:**
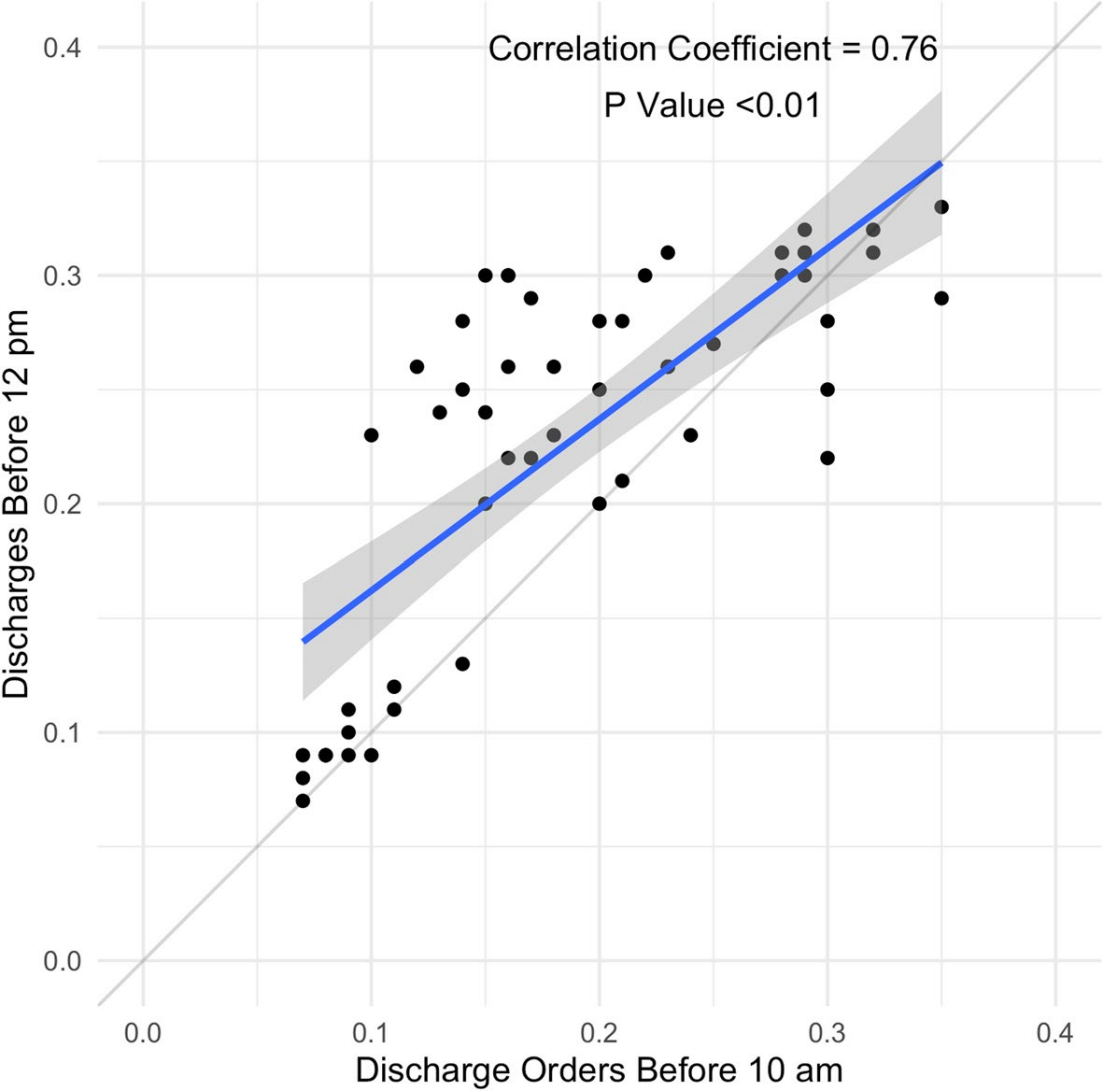
Correlation of monthly discharge order proportion (before 10 am) with monthly discharges before noon proportion

A Pearson correlation test was run on the monthly DCOB and DBN to confirm the relationship between the two variables. The resulting output showed a strong and significant correlation between the two variables (*r* = 0.76, *p* < 0.001) (see Fig. 3).

## Discussion

Our study demonstrates sustained improvement in discharge before noon from 9.45% to 26.6% over a 41-month period. This improvement was accomplished without negatively impacting the RA-LOS, RA-readmission rate, or RA-mortality. The improvement in DBN was driven by an increase in discharge orders placed before 10 am; notably, we found a strong correlation between DCOB and DBN. Providers continued to emphasize entering discharge orders early even when missing the 10 am target. Nursing and case management continued to strive for DBN even when discharge orders were placed between 10 am and noon, and this is reflected in our DBN results for phase 1 and phase 2, which show higher DBN rates than the DCOB.

Other academic centers have also been successful with early discharge interventions for adult medicine inpatients. Wertheimer et al. showed results of 38% discharge before noon on a single floor with 70 patients [7]. Kane et al. showed improvement up to 24% discharge before noon on medicine services for 11 months [17]. While these studies have shown improvement in DBN rates, they have been implemented on a smaller number of patients over shorter time periods compared to our study. Our DBN study spans 41 months and covers 14 medicine teams with a combined census of up to 250 patients dispersed across 7 floors in 3 geographically distinct towers.

The complexity of the discharge process underlies the difficulty of achieving early discharges. DBN requires alignment of multiple overlapping and sequential processes among multidisciplinary stakeholders. Several factors can lead to delays in discharge, such as delays in clinical decision making, pending results, care coordination/placement, ineffective communication between the teams/providers, therapy delays, and other logistics [10, 18, 19]. We found that these challenges were amplified during major surges of patients with COVID-19. Over half of the study period occurred during the worst part of the COVID pandemic. Some unique issues posed during the height of the COVID-19 pandemic included difficulty obtaining home oxygen, isolation requirements complicating disposition and transportation, and lack of appropriate and timely follow-up. We found that an iterative, multidisciplinary approach involving key stakeholders, including nursing, case management, and physicians/APPs, helped address challenges posed by COVID-19 and enabled us to maintain high DBN rates throughout the pandemic [5–7, 10, 17]. One example is a pivot to the utilization of EMR secure chat-based communication instead of in-person multidisciplinary rounds. This helped us maintain interdisciplinary teamwork even when social distancing was required at times of peak COVID activity.

We deployed structured twice daily multidisciplinary rounds involving nursing, care coordination, and physicians/APPs. While other institutions have utilized once-a-day multidisciplinary rounds as a part of their efforts to improve DBN [7, 17], we successfully incorporated the following components to optimize the efficiency of multidisciplinary rounds: (1) a script to focus the discussion for each patient, (2) identification of patients using a color code for discharge readiness, (3) a checklist of tasks for discharge readiness and 4) an escalation pathway for barriers to discharge.

Another novel element in the success of discharge before noon was the initiation of team-based, as opposed to unit-based, care coordination. Given the lack of geographic localization on our services, having the same case manager/social worker/discharge expediter covering a medicine team made communication efficient and served as an effective avenue for reporting and escalating issues causing delays in care. Hemali et al. reported improvement in discharge orders before noon, LOS, and readmissions when a team case manager was involved in multidisciplinary rounds [6]. Once their pilot project concluded and case management returned to the unit-based model, there was a regression of discharge orders to the pre-pilot mean. Similarly, we found regression when our care coordination returned to the unit-based model in June 2021. We were able to mitigate this issue by initiating secure chat-based communication between providers, nursing, and case management in phase 3.

We encountered several barriers to sustaining high rates of DCOB and DBN over two years. These include time investment in flash rounds with increasing patient census, need for frequent orientation of new faculty physicians, APPs, and residents to the process, provider fatigue and turnover among case management, nursing, and providers. We have increased the number of hospital medicine teams to address increasing patient census per team, and designed a structured shadowing program for new faculty and APPs that includes experiential learning and group sessions on the importance of multidisciplinary rounding [20]. We continue to encourage the placement of discharge orders before 10 am by sharing weekly DCOB and DBN metrics with providers, including residents, APPs, and faculty physicians. For the teaching teams, awards are given to the team with the highest number of early discharges. Such interventions were important motivators to maintain momentum on the project. In addition, initial and sustained engagement of hospital leadership, along with standardized performance packet feedback on quality metrics for providers has helped to cultivate a culture of quality [21]. To facilitate ongoing commitment by all stakeholders, we have a standardized script for flash rounds to keep them brief, and periodic audits to ensure efficiency and quality of the rounds.

While we cannot causally attribute the improvement in RA-readmission to our intervention, it is possible our project had an impact on readmission rates. When discharging early in the day, the full team of daytime providers, nursing, case management, physical and occupational therapists, and pharmacy is available to address any last-minute issues that arise with discharge planning. With our daily flash rounds, patients benefit from an interdisciplinary discussion to ensure a complete assessment of discharge readiness with input from all stakeholders. It is also possible that other concurrent initiatives by the hospital may have impacted this metric during our project. Examples of such initiatives include pharmacy medication reconciliation at admission and a congestive heart failure nurse navigator program for transitions of care management [22, 23].

Our study has limitations. First, many interventions were implemented, so it is difficult to determine the relative impact of each intervention. Second, our study does not include a control group. While it is possible that the improvement seen in DBN reflects secular trends, we think this is highly unlikely. We observed a significant and sequential increase in DBN with the introduction of each set of interventions. Conversely, we saw a decline in DBN in June 2021 as flash rounds were put on hold due to the pressures of COVID-19. It is also unlikely that the increase in DBN was a result of interventions outside of our project, since all the interventions to improve early discharges at our institution were implemented as part of this project. Third, our intervention including flash rounds primarily occurred during the week due to limitations in staffing for weekend multidisciplinary rounds. Finally, this study was at a single institution. However, our program is structured similarly to other hospitalist programs at other large academic medical centers. The successful implementation of our interventions and adaptive measures undertaken to surmount a myriad of challenges over the extended period of this study can serve as a helpful framework for other institutions.

### Supplementary Information

**Supplementary Material 1.**

**Supplementary Material 2.**

**Supplementary Material 3.**

**Supplementary Material 4.**

## Data Availability

The datasets generated and/or analyzed during the current study are not publicly available but are available from the corresponding author upon reasonable request.
