# Supplementary material for: Increasing and sustaining discharges by noon – a multi-year process improvement project
Source: BMC Health Serv Res. 2024 Apr 17;24:478. doi: 10.1186/s12913-024-10960-x (PMC11025149; doi:10.1186/s12913-024-10960-x)

CHECKLIST FOR SAFE AND TIMELY DISCHARGES

ADMISSION DAY

Assess during H&P:

- ☐ Mobility: "How do you get around at home? Do you need a cane or walker? Do you feel weaker?"
- ☐ Placement: "What's your living situation like? Do you live with family or have support?"
- ☐ Medication Reconciliation and Adherence (affects discharge meds, especially safety and re-admissions): "Does anyone help you take your medications or do you manage them yourself?"

DURING HOSPITALIZATION

Interprofessional consults as soon as medically appropriate (anticipate DME, facility placement, or otherwise not at baseline):

- ☐ PT/OT
- ☐ SLP
- ☐ Dietician/Diabetes Education
- ☐ Wound Care
- ☐ Respiratory Therapy

Daily re-evaluation of any pertinent needs:

- ☐ Outpatient follow-up appts (e.g., PCP, specialty appts, funding needs)
- ☐ BM/Urinary Retention/Foley
- ☐ IV meds (d/c if not needed)
- ☐ Consult notes: place orders based on recommendations (including medical specialties interprofessional services)
- ☐ Procedures: discussed with patient and orders placed (NPO, hold dvt ppx, INR, plts)
- ☐ VMT/Sitter/Restraints (d/c if not needed)
- ☐ Outpatient IV ABX: Place IV ABX order and PICC
- ☐ Home O_2: Evaluation and place order if needed

DAY OR TWO BEFORE DISCHARGE

Review medical needs:

- ☐ Acute medical conditions resolved/stabilized (this is what we're exceptionally good at!)
- ☐ Specialty/Subspecialty medical recommendations (check-in and tell them plan to discharge as courtesy)
- ☐ Vital signs and lab abnormalities
- ☐ Pending procedures (e.g., dialysis, imaging): schedule earlier or in the morning

Coordinating with CM/SW and Patient

- ☐ Set patient expectations kindly: "You seem to be getting better and might be ready to discharge in the morning tomorrow if things keep getting better"
- ☐ Double-check interprofessional needs: DME, home health, other supplies
- ☐ Transportation (e.g., family to pick up, bus ticket, cab voucher, transport time to facility)
- ☐ OOH DNR/DNI (if going to facility or transporting by ambulance)

Medication reconciliation:

- ☐ Evaluate medication needs: PRN meds, triplicate meds, and chronic meds needing refills
- ☐ Send partial medication reconciliation to pharmacy (to avoid overwhelming pharmacy at peak times and prevent delays; also will find out if a certain medication is not covered by insurance and have time to prescribe alternative)

DISCHARGE DAY

- ☐ See patient and evaluate if medically clear for discharge (ask attending if they can see before rounds)
- ☐ Send remaining medications to complete reconciliation
- ☐ Place discharge orders and write discharge summary

DISCHARGE PROCESS MAP

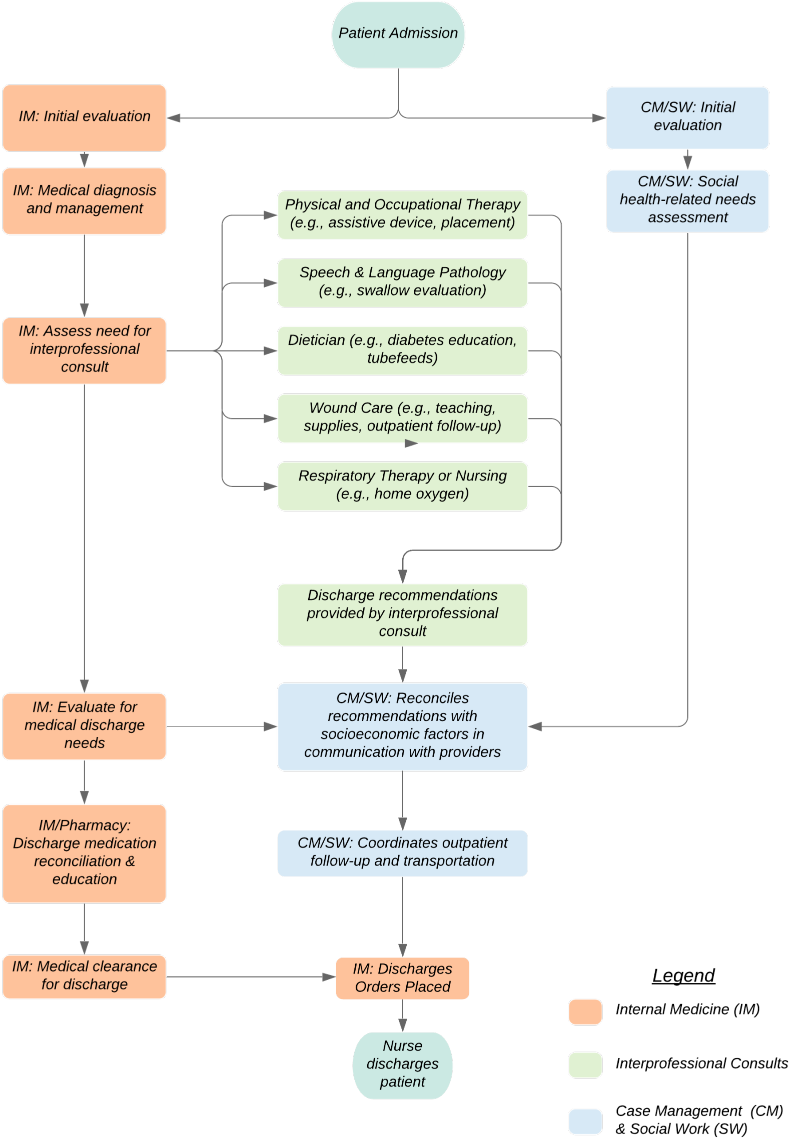

Supplement: Supplementary file 1 — Supplementary Material 1. [file 12913_2024_10960_MOESM1_ESM.pdf]
