# Supplementary material for: Increasing and sustaining discharges by noon – a multi-year process improvement project
Source: BMC Health Serv Res. 2024 Apr 17;24:478. doi: 10.1186/s12913-024-10960-x (PMC11025149; doi:10.1186/s12913-024-10960-x)
Supplement: Supplementary file 2 — Supplementary Material 2. [file 12913_2024_10960_MOESM2_ESM.pdf]

PROVIDER'S CHECK LIST

Please check below when you are planning to discharge a patient

YELLOW DAY – 24 HOURS BEFORE DISCHARGE

Answer the following questions to guide when planning. Have you?

Ordered an anticipate discharge?	
Ordered vaccines?	
Started the medication reconciliation?	
Does your patient need DME? If so, have you ordered it? Check PT notes.	
Does your patient need prescription for narcotics? If so, did you obtain triplicates?	
Order Home antibiotics as applicable?	
Order all the out patient referrals as **OP Consults upon discharge?**	
Talk to patient and family about the discharge plan?	
Does your patient need lab before going home? If so, please order to have it done at 10 PM so this can be process early as appropriate. If patient needs labs in the morning before discharge- order it at 3 AM for early results. Order to notify you for results.	
Sign the work/school excuse for patient with restrictions and extended days off. Have the RN print this for you and just sign it.	

If one of these questions answer is no, please check with your care coordinator (Case manager, Social worker)

GREEN – DAY OF DISCHARGE

Before discharging your patient, please answer the following questions. Have you?

Order for discharge by 0900?	
Complete medication reconciliation?	
Obtain triplicate for narcotics from your faculty?	
Completed discharge summary if patient going to another facility?	
Speak to the patient and family about discharge?	
Fax /e-scripts the prescriptions to patient preferred pharmacy?	
Check- if out patient referrals processed by Access Plus?	
Have you reviewed discharge meds with your faculty?	
