# Supplementary material for: Increasing and sustaining discharges by noon – a multi-year process improvement project
Source: BMC Health Serv Res. 2024 Apr 17;24:478. doi: 10.1186/s12913-024-10960-x (PMC11025149; doi:10.1186/s12913-024-10960-x)
Supplement: Supplementary file 3 — Supplementary Material 3. [file 12913_2024_10960_MOESM3_ESM.pdf]

DATE:____________________ RM NO:_________ PT INITIAL ___________

INSERT THIS INSIDE SIGN HOLDER ON FRONT OF PATIENTS DOOR

RNS and Techs: Initial this GREEN form as you complete it. Please write NA for any information that is not applicable for your patient TECHs: Once the form has been completed by the RN, please turn in the completed form to the charge nurse upon discharge to track the cause of delay.

GREEN DAY POWER THROUGH

	**RN Initial**
Discharge order is written and complete	
Confirm that discharge instructions are completed, documented and printed (AVS)	
Contact designated ambulance/ wheelchair van/ family service for transport for pick up schedule once order for discharge is receive.<br>TIME CALLED_________ ETA_____________	
Verify care measures education are completed and documented- Vaccines, CHF, DM, VTE, stroke, Lovenox teaching, insulin	
Discontinue lines – IV , central lines as applicable (except for patient going home with lines)	
Call Meds To Beds to verify medications are ready to be delivered	
Notify charge nurse for barriers:____________________________	
Call report to facility if applicable	

**DAY OF DISCHARGE – GREEN- TECH ROLE**	**TECHS INITIALS**
Confirm that belongings, clothing and shoes are obtained	
Confirm that patient has ordered early breakfast/ lunch. Notify dietary supervisor for to go @ 8-0368	
Confirm that shower/ bath completed	
Place EPIC transport order for TCC – can call unit clerk for assistance	
Strip the room and empty suction canisters, chest tube drains and other drains place in biohazard room.	
Dirty equipment place in soiled utility room	
Patient's drawer empty	
TIME OF DISCHARGE_____________________________	
