# Supplementary material for: Increasing and sustaining discharges by noon – a multi-year process improvement project
Source: BMC Health Serv Res. 2024 Apr 17;24:478. doi: 10.1186/s12913-024-10960-x (PMC11025149; doi:10.1186/s12913-024-10960-x)
Supplement: Supplementary file 4 — Supplementary Material 4. [file 12913_2024_10960_MOESM4_ESM.pdf]

DATE:___________________ RM NO.__________ PT INITIAL __________

PLACE THIS OUTSIDE THE PATIENT'S ROOM FOR COMMUNICATION

***RNs and Techs: Initiate this yellow form as you identified your patient as anticipate discharge in 24 hours. Please initial as you complete it and write NA for any information that is not applicable for your patient

** TECHs: Once the form has been completed by the RN, please turn in the completed form to the charge nurse. Then initiate the green form immediately.

YELLOW DAY POWER THROUGH

24Hrs prior to discharge- RN Role	RN Intials
Order for vaccine received from MD	
Home Health orders entered & DME delivered – O2, CPAP, etc.	
Lovenox, Insulin, Coumadin, stroke, CHF, DM, etc. teaching completed	
Wound Vac switched to portable	
Review/ completed POC/ Education in Epic- Care Measures teaching – Stroke, CHF, Pneumonia, VTE	
Tube feedings formula and supplies secured – teaching completed	
Work/ school excuse obtained or FMLA form	
Walk of life completed	
Transportation confirmed before 12: which Taxi, Bus, POV, Ambulance, WC van	
PT/OT/ ST recommendations followed and completed	
Ensure that labs are drawn by 2200 /0300– call for corrections if needed	
Notify charge nurse for pending procedures	
Notify charge nurse for barrier	

NIGHT BEFORE DISCHARGE

TRANSITIONING INTO GREEN (TECH ROLE)	TECH INITIAL
Check with UC if patient has valuables secured in the hospital	
All belongings packed and secured	
Bathing/ shower given	
Confirm that patient has breakfast ordered	
Notify charge nurse for barriers	
